# Linehan’s biosocial model applied to emotion dysregulation in autism: a narrative review of the literature and an illustrative case conceptualization

**DOI:** 10.3389/fpsyt.2023.1238116

**Published:** 2023-09-29

**Authors:** Doha Bemmouna, Luisa Weiner

**Affiliations:** ^1^Faculté de Psychologie, Université de Strasbourg, Strasbourg, France; ^2^Département de Psychiatrie Adulte, Hôpitaux Universitaires de Strasbourg, Strasbourg, Alsace, France

**Keywords:** autism spectrum condition, emotion dysregulation, self-harm, suicidality, Linehan’s biosocial model, etiology

## Abstract

Emotion dysregulation (ED) is a transdiagnostic difficulty prevalent in autism spectrum condition (ASC). Importantly, recent research has suggested that ED is involved in self-harm and suicidality. Pre-existing models on the etiology of ED in ASC focus mainly on biological factors to ASC features, such as sensory sensitivities, poor flexibility, and sensitivity to change. However, although psychosocial factors seem to play a role in the emergence of ED in ASC as well (e.g., childhood maltreatment and camouflaging), there is a lack of a comprehensive model conceptualizing biosocial factors involved in ED in autistic people. Linehan’s biosocial model (1993) is one of the leading etiological models of ED in borderline personality disorder (BPD). It conceptualizes ED as emerging from transactions between a pre-existing emotional vulnerability in the child and an invalidating developmental environment. Beyond its clinical relevance, Linehan’s model has gathered empirical evidence supporting its pertinence in BPD and in other psychiatric disorders. Although ASC and BPD are two distinct diagnoses, because they may share ED, Linehan’s biosocial model might be useful for understanding the development of ED in ASC. Hence, this article aims to provide an application and extension of Linehan’s model to conceptualize ED in ASC. To do so, we conducted a narrative review of the literature on ED and its underlying factors in ASC from a developmental perspective. To investigate the pertinence of the biosocial model applied to ED in autistic people, we were interested on data on (i) ED and its behavioral correlates in ASC, in relation to the biosocial model, (ii) the potential biological and psychosocial correlates of ED in ASC and (iii) the overlapping difficulties in ASC and BPD. Finally, to assess the pertinence of the model, we applied it to the case of an autistic woman presenting with ED and suicidal behaviors. Our review and application to the case of an autistic woman suggest that ED in ASC encompasses factors related to both biological and psychosocial risk factors as conceptualized in the BPD framework, although in both domains ASC-specific factors might be involved.

## Introduction

Emotion dysregulation (ED) refers to emotional experience and/or expression that interferes with appropriate goal-directed behavior (1). ED has been widely studied in borderline personality disorder [BPD; (2, 3)]. Linehan’s biosocial theory (1993), one of the leading etiological models of BPD, places ED at the core of the disorder. Linehan’s theory conceptualizes ED as emerging from transactions between emotional vulnerability and an invalidating developmental environment (4, 5). Emotional vulnerability refers to biological factors with a genetic basis evidenced by disruptions in the emotional system involving different brain areas (e.g., prefrontal regions and amygdala) (6). Linehan’s theory has subsequently added temperamental impulsivity as an additional risk factor for BPD (4). Emotional vulnerability results in dysfunctions in three dimensions: (a) emotional hypersensitivity (i.e., low threshold for emotional reactions), (b) hyperreactivity (i.e., increased change in emotional intensity and extreme reactions), and (c) a slow return to emotional baseline (i.e., long-lasting emotional reactions) (6, 7). Invalidation, on the other hand, refers to the inadequate responses of the environment to the emotional needs of the child (5). It may occur through the neglect, minimization or punishment of the child’s emotional experience, but also through physical and sexual abuse (4, 8). According to Linehan’s theory, in people presenting with emotional vulnerability, early invalidation may result in maladaptive coping (i.e., self-harm with or without suicidal intent) when they are faced with difficult emotions (9, 10). An important corollary of this biosocial perspective is that, during their development, people with BPD did not learn the adaptive skills to regulate their emotions effectively. Hence, they may display rigid and pervasive dysfunctional strategies that were involuntarily targets of operant conditioning (e.g., “mom listens to me and is nice when I cut myself”; “dad says that it is stupid to cry”) or modeling (5). Therefore, as adults, people with BPD lack the skills to regulate their emotions, as they were taught that emotional reactions are not to be trusted (i.e., they self-invalidate) and that emotions are dangerous and should be escaped or avoided (e.g., using crisis behaviors or emotional avoidance) (5). Importantly, Linehan developed dialectical behavior therapy (DBT), the treatment targeting ED with the most empirical support, based on this model (11, 12).

Some empirical studies have tested Linehan’s model in BPD [e.g., (13, 14)]. Although it is not consensual, the model has amassed considerable evidence in its support. For instance, Reeves et al. (14) found that emotional vulnerability and ED were substantially associated with BPD symptoms, with ED mediating the relationship between emotional vulnerability and BPD symptoms. In addition, Carpenter and Trull (2) gathered findings supporting the role of biological factors such as emotion sensitivity and lability in the emergence of BPD. Interestingly, studies have supported the role of emotional hypersensitivity and slow return to baseline but not hyperreactivity in the emotional vulnerability found in people with BPD (6, 7). Regarding invalidation, Reeves et al. (14) found that parental invalidation in childhood does not predict BPD, while other findings have come to opposite conclusions (15–17), particularly concerning the involvement of maternal invalidation (18). Beyond the association between parental invalidation and BPD, cultural and intra-individual factors seem to be involved in the emergence of BPD. For instance, Keng and Soh (18) found that the association between self-reported maternal invalidation and BPD was moderated by two cultural factors: self-construal (i.e., the extent to which the self is defined independently of others or interdependently with others) and conformity to norms (18). In addition, Keng and Wong (16) showed that low levels of self-compassion were associated with BPD independently of parental invalidation. Finally, regarding the transaction between emotional vulnerability and invalidation, some studies support the transaction (4, 19) while others do not (13).

ED is strongly associated with BPD (3). However, several recent findings suggest that ED is a transdiagnostic mechanism of psychopathology (20–24). Although Linehan’s model has not been directly studied outside BPD, findings support the involvement of biological vulnerability and invalidation in the emergence of ED in various psychiatric disorders (25), including trait impulsivity (26), and childhood maltreatment (linked to invalidation) (27–29).

In autism spectrum condition (ASC),^1^ there has been a growing interest in overlapping difficulties with BPD, including ED (32, 33). Indeed, there is a considerable overlap in the diagnostic criteria for ASC and BPD (e.g., difficulties in social interactions) (34), which increases the risk of misdiagnosis, especially in women (35, 36). The high prevalence of ED in autistic individuals [e.g., between 50 and 60% in autistic youth; (37–39)] and its association with self-harm and suicidal behaviours (40, 41) may be an additional source of misdiagnosis with BPD (42). Indeed, autistic people presenting with ED and self-harm are reported to be at greater risk of being misdiagnosed with BPD since these difficulties are strongly associated with BPD (36, 42). It should also be noted that ASC and BPD can co-occur (34), with the co-occurrence being linked to higher suicidality than in BPD or ASC alone (43–45).

Few studies have investigated the etiological factors involved in ED in ASC, and most have focused exclusively on the role of ASC-related factors [e.g., (38, 39, 46)]. Thus, to our knowledge, no studies have attempted to conceptualize ED in ASC in relation to Linehan’s model. This is of special relevance given the emerging interest in DBT to treat ED in ASC (47, 48). Indeed, recently, DBT has been found to be feasible and acceptable in autistic adults without intellectual disability (49, 50). In those with self-harm and suicidal behaviours, initial evidence suggests that DBT is effective in reducing ED (49). Nevertheless, to improve the pertinence of DBT to autistic individuals, it is of the utmost importance to provide treatments that consider the specific features potentially involved in ED in this population (51).

Case formulation is central to effectively implement behavioral treatments (52). Linehan’s biosocial model provides a theoretical framework to inform case formulation when treating clients with BPD. However, it is still unknown whether it might also apply to autistic adults. This is crucial since DBT is in its early stages in ASC and that ED in autistic people is still poorly understood (32).

This article aims to provide an application of Linehan’s model to conceptualize ED in ASC. To do so, we conducted a narrative review of the literature on ED and its underlying factors in ASC across the lifespan. Indeed, narrative reviews are well suited to address research questions with a broad scope to draw conclusions and generate areas for future research questions (53, 54). To investigate the pertinence of the biosocial model applied to the ED found in autistic people, we were interested on data on (i) ED and its behavioral correlates in ASC, in relation to the biosocial model, (ii) the potential biological and psychosocial correlates of ED in ASC and (iii) the overlapping difficulties in ASC and BPD. Finally, to assess the pertinence of the model, we applied it to the case of an autistic woman presenting with ED and suicidal behaviours.

Our review was conducted using PubMed, Medline Ovid SP and PsycINFO search engines. Articles had to meet the following inclusion criteria: (a) articles published after 2000, (b) articles in English, (c) articles published in a peer-reviewed journal (d) articles interested in autistic individuals without intellectual disability and/or individuals with BPD. Given that our approach was developmental, we included articles on ED and its correlates in both youth and adults with these diagnoses. Hence, we specify throughout our review whether the findings relate to youth or adults. Our articles research paired keywords were the following ones: “Emotion dysregulation,” “Emotion regulation,” “Emotion,” “Emotional reactivity,” “Autism,” “Adults,” “Children,” “Youth,” “Adolescents,” “Borderline personality disorder,” “Impulsivity,” “Impulsiveness,” “Self-harm,” “Non-suicidal self-injury,” “Suicidality,” “Suicide,” “Linehan’s biosocial model,”” Linehan theory,” “Emotional vulnerability,” “Invalidation,” “Trauma,” “Adverse events,” “Bullying,” “Autistic camouflaging,” “Emotional scaffolding,” “Predictors,” “Correlates,” “Aetiology,” “Etiology.” To ensure the quality of our narrative review, we referred to the six criteria listed in the Scale for the Assessment of Narrative Review Articles [SANRA; (55)].

For the illustrative case conceptualization, we used the client’s quantitative and qualitative data. The client provided informed consent for the use of her data and participated in building and writing the case conceptualization. The use of personal data was approved by the University of Strasbourg research ethics board (Reference: CE-2022-138).

### Emotion dysregulation in ASC

Recent research suggests that autistic people are more likely to develop ED than the general population (32, 33, 39). In fact, studies have shown fewer emotion regulation abilities and greater maladaptive strategies (e.g., rumination, avoidance) in autistic youth compared to their non-autistic peers (32, 56). Although ED has been mostly studied in autistic youth, it also concerns adults (57). Similar to findings in the general population (58), autistic women appear to present with greater ED than autistic men (59–61).

ED is not a diagnostic criterion for ASC (62), but given its high prevalence in autistic people, some researchers have questioned whether it should be added to ASC core features (39, 40, 63). Indeed, ED has been found to be highly associated with autistic core features (39, 51). Among them, restricted and repetitive behaviours (RRBs) in particular have been found to be the strongest ED predictor in autistic people (39, 64), suggesting that RRBs might contribute to ED, possibly through inhibitory dyscontrol (i.e., executive dysfunction) (39). Indeed, effective emotion regulation relies on inhibitory control and cognitive flexibility, which enables the use of context-dependent strategies (65). This is especially the case for emotion regulation skills that require increased adaptability, such as problem solving and cognitive reappraisal (65). Executive dysfunction might thus interfere with this ability (32, 51, 66), and lead to the increased use of maladaptive emotion regulation strategies (e.g., rumination, avoidance, suppression) but also RRBs (38). However, other findings indicate that RRBs rather stem from ED (39, 64, 67), since one quarter of these behaviours appear in reaction to emotional triggers (67). Irrespective of the direction of the relationship between ED and RRBs, recent data suggest that self-harming behaviours in ASC are similar to those seen in the general population (68). Therefore, contrary to past research suggesting that self-harming behaviours are part of RRBs in autistic people (69, 70), self-harming behaviours might actually be distinct from RRBs. Indeed, recent studies suggest that self-harming behaviours are used by autistic people to regulate painful emotions, particularly low-energy affective states like sadness and high-energy affective states like anger and anxiety (41, 71). By contrast, unlike self-harming behaviours, RRBs may serve the function of sensory stimulation and are primarily characterized by their automatic and stereotyped nature (62).

There is a lack of consensus on whether ED is a core problem in ASC or whether it is stems from co-occurring disorders (38), as co-occurring mental health issues (e.g., anxiety and depression) are prevalent in ASC (72, 73) and that ED is a transdiagnostic difficulty (23). However, a growing number of studies suggest that co-occurring disorders result from preexisting ED in ASC, suggesting that ED predisposes to the emergence of psychiatric disorders especially in adults (40, 57, 74). Given that few studies with a longitudinal design have focused on ED in ASC [e.g., (64)], the direction of the relationship between ED and psychiatric disorders in ASC has not been yet been fully elucidated.

In ASC, ED has been associated with dysregulated behaviours (e.g., meltdowns, outbursts) (38, 75). Similar to BPD (76), recent studies suggest that ED is involved in self-harm with or without suicidal intent in ASC (40, 41, 71, 77). However, only recently research has started to highlight the high prevalence of self-harm (71, 78, 79) and suicidality in ASC (80, 81). A meta-analysis revealed a prevalence of 42% of self-harm in autistic people, irrespective of age and the presence of intellectual disability (79). Some findings suggest that the characteristics of these behaviours are similar to those found in the general population (68) and might be used by autistic people to regulate painful emotions, particularly low-energy affective states like sadness and high-energy affective states like anger and anxiety (41, 71). Moseley et al. (41) suggest that self-harm in ASC may also have other functions: i.e., self-punishment, deterrence from suicide, sensory stimulation and/or social communication.

Regarding suicidality, reviews have reported prevalence rates in ASC between 10 and 50% (82, 83). High suicidality rates have been reported in both autistic youth (84) and adults (77, 85), with adults without intellectual disability, especially women, being at the highest risk of dying by suicide among the autistic population (85–88). In relation to ED, Conner et al. (40) found that elevated ED was associated with increased suicidal behaviours in autistic youth. Some findings support a strong association between self-harm and suicidality, suggesting that autistic adults may develop capability for suicide through self-harm (78, 89).

Few studies have investigated factors contributing to the heightened rates of ED and suicidality in autistic women relative to autistic men (59, 61). In addition to an increased anxiety (59), recent findings point to an increased use of camouflaging in autistic women contributing to elevated distress and risk of suicidality (90, 91). In addition, autistic women, especially those without intellectual disability, are at higher risk of late diagnosis than men (92), which increases their exposure to invalidation and the pressure of exhibiting socially appropriate behavior (93). Although autistic women might be more likely to mask their social difficulties, these difficulties (e.g., identifying others’ intentions) persist even though they are less visible to others, making them more vulnerable to the societal invalidation toward women, particularly sexual violence (94, 95). These findings suggest the need to pay special attention to mental health in autistic women, especially regarding ED and suicidality.

As in BPD (96), Conner et al. (33) pointed to ED as a risk factor for the use of psychotropic medication, emergency services and psychiatric hospitalizations among autistic people. ED also contributes to impairments in adaptive functioning in ASC in childhood (97, 98) and adulthood (38).

Overall, findings support the implication of both emotional vulnerability and invalidating experiences in the development of ED in ASC [e.g., (40, 99–101)]. However, to our knowledge, no studies investigated the transactional relationship between the two components in this context.

### Biological correlates of ED in ASC

#### Biological vulnerability to ED

Numerous studies have linked autistic features, including peculiarities found at the emotional level, to the atypical brain development in ASC (38, 102). Indeed, neuroimaging findings show an atypical neural functioning underlying impaired emotion regulation in ASC (101, 103–105). For instance, in autistic adults without intellectual disability, Richey et al. (101) reported a hyporegulation of key brain regions involved in effortful emotion regulation (i.e., decreased ability to enhance the nucleus accumbens’ activation and to lower the amygdala’s activation) while engaging in cognitive reappraisal compared to non-autistic adults (101). Autistic adults also showed decreased dorsolateral prefrontal cortex activation (dlPFC) during the task, another brain region involved in goal-directed processes (101). By contrast, some previous findings showed a hyperactivation in this region in ASC, suggesting a potential compensatory activation to overcome cortical inefficiency during effortful emotion regulation (106). Moreover, using functional magnetic resonance imaging (fMRI), Mazefsky et al. (103) found a longer lasting brain activity in areas involved in sustained emotional information processing (i.e., insula, pulvinar and dlPFC), akin to rumination, in autistic youth compared to non-autistic peers. These regions have been shown to be involved in ED in conditions other than ASC (107, 108). This suggests an atypical neural activity behind the tendency to ruminate in ASC, which is a maladaptive emotion regulation strategy prevalent in autistic people (103).

Despite the paucity of studies, some findings support the involvement of the three dimensions of emotional vulnerability (i.e., hypersensitivity, hyperreactivity and slow return to emotional baseline) in ASC. For instance, Lassalle et al. (109) found that autistic individuals were hypersensitive to fear stimuli with a significantly higher activation of the amygdala than non-autistic individuals. Sensory hypersensitivities have also been linked to increased psychophysiological arousal and increased anxiety in autistic people (40, 110, 111). Regarding hyperreactivity, the majority of findings have reported an increased physiological response to emotional stimuli in autistic individuals compared to non-autistic individuals [e.g., (105, 112)]. However, few other studies have rather supported equivalent physiological arousal to emotional triggers between autistic and non-autistic individuals (39, 113). It is noteworthy that discrepant results have also been reported in BPD regarding physiological hyperreactivity (6, 7). Finally, there are findings in support of the long-lasting nature of emotional arousal in autistic children, evidenced by the prolonged duration of cortisol secretion following a stressor compared to non-autistic peers (114).

#### Challenges related to ASC core features that contribute to ED

A growing body of evidence suggests a link between core ASC features and ED, with the higher the autistic traits, the higher the ED (39, 51, 71, 115). Relatedly, Samson et al. (39) found that interventions enhancing emotion regulation skills in autistic children improved not only ED but also difficulties related to ASC features, which indirectly supports the link between ED and autistic traits.

**Figure 1:**
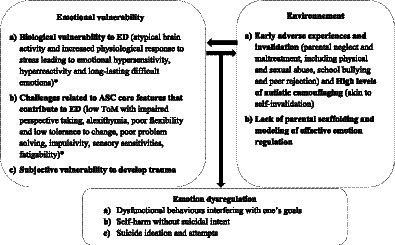
Linehan’s model application to ED in ASC. * Factors included in Mazefsky and White’s model (2014).

Effortful emotion regulation is a deliberate process of self-regulation (116). Thus, due to the additional daily challenges linked to ASC-related difficulties (e.g., executive dysfunction, social interaction difficulties) and subsequent anxiety and fatigue, it is crucial to acknowledge that emotion regulation may come with increased cost for autistic people (39, 51, 117, 118). Beyond this increased load, ASC-related difficulties might directly interfere with effective emotion regulation (39, 51). Therefore, the “emotional vulnerability” component of the biosocial model that we propose includes the contribution of ASC-features previously acknowledged in Mazefsky and White’s model (2014) and integrates recent findings (Figure 1).

Effortful emotion regulation requires accurate identification of key aspects of the situation to use appropriate strategies (65). However, difficulties with social skills in ASC (62), particularly due to theory of mind (ToM) peculiarities, may interfere with effective emotion regulation (51, 119). However, it is important to highlight that the ToM peculiarities are neither specific to nor systematic in ASC (120). Additionally, effortful emotion regulation requires identifying one’s emotional experience to be able to modulate it (121). Yet, alexithymia, which refers to the difficulty in identifying and expressing one’s emotions, is common in ASC (122), limiting insight into one’s own emotions and thus preventing their deliberate modulation (38, 51). In autistic adults, alexithymia has been found to predict self-harm, particularly when experiencing high-energy states (i.e., anger, anxiety) (41). In autistic women in particular, alexithymia has been found to be related to ED, irrespective of BPD traits (123). Together, these difficulties could explain why autistic people may react impulsively to emotional triggers with a lack of goal-directedness (38).

Furthermore, effective emotion regulation relies on cognitive flexibility, which enables the use of context-dependent strategies (65). This is especially the case for skills that require increased adaptability, such as problem solving and cognitive reappraisal (65), and unfamiliar situations that also trigger the change-related anxiety common in ASC (51, 62). Poor cognitive flexibility might thus interfere with this ability in autistic people (32, 51, 66), and lead to the overuse of maladaptive emotion regulation strategies (e.g., rumination) or to the rigid use of adaptive emotion regulation strategies that do not fit the ongoing situation (e.g., distress tolerance skills) (38, 124). Consistent with this view, Aldao and Nolen-Hoeksema (125) suggested that individuals might develop a ‘default’ regulatory approach that interferes with the ability to use new and more adaptive strategies, such as reappraisal. This might be particularly true for autistic people, due to cognitive flexibility difficulties and change-related anxiety. Moreover, RRBs have been found to be the strongest predictor of ED in ASC (39), with one quarter of them appearing in reaction to emotional triggers (67). This points to the difficulty in inhibiting automatic behaviors in ASC, which also interferes with goal-directed behaviors and flexible emotion regulation (38, 39). Interestingly, emotional vulnerability, particularly the increased and sustained physiological activation following emotional triggers, possibly interfere with both cognitive and behavioral control in ASC (126). Thus, increased emotional vulnerability might promote automatic emotional responses in autistic individuals, which interferes with effortful emotion regulation (126). Moreover, it is noteworthy that ADHD, characterized in its hyperactive dimension by impulsivity, frequently co-occurs with ASC, including in adults (e.g., (127) found a prevalence of 33.3%). Therefore, if present, ADHD co-occurring features are likely to contribute to emotional difficulties (128).

Sensory sensitivities, which are common in autistic people (62), have also been reported to be significantly related to ED in ASC (39–41, 51). In a sample of autistic adults without intellectual disability, Moseley et al. (41) found that sensory sensitivities were a strong predictor of self-harming behaviors along with alexithymia, anxiety and depression (41). Importantly, in autistic youth, some studies have suggested that sensory sensitivities were the strongest and single predictor of self-harm (129). This association may be due to the distress reported by autistic people when experiencing intense sensory discomfort (130).

### Psychosocial risk factors contributing to ED in ASC

#### Early adverse experiences and invalidation

Autistic people are at heightened risk of experiencing adverse childhood events (131–133), particularly autistic girls (134, 135). This increased exposure to adverse experiences can be understood through the double-empathy theory, which highlights difficulties of reciprocity and mutuality between autistic and non-autistic people due to a lack of mutual understanding of each other’s subjective experience (136, 137). Hence, on the one hand, autistic individuals may face challenges in understanding and fitting into a “non-autistic” environment due to their particularities, and, on the other hand, the environment around them may contribute to their exclusion by failing to understand their atypical functioning and needs (138). As a result, from childhood onwards, autistic people are more likely to be rejected and maltreated, both within the family and in the wider community.

Moreover, some studies also highlight an increased vulnerability to be detrimentally affected by adverse events in ASC, with a wider range of events acting as possible catalysts for trauma (e.g., “sensory trauma” and major changes) (139, 140), supporting the hypothesis of a transactional relationship between biological and social factors in the emergence and maintenance of ED in autistic people. Adverse experiences in autistic children are associated with co-occurring disorders and/or the worsening of ASC-related difficulties in childhood (133, 141) and in adulthood (e.g., mood and anxiety disorders, PTSD) (131).

Autistic children may experience different forms of adverse events. First, autistic children, including those without intellectual disability, are at heightened risk of maltreatment, particularly physical neglect, and abuse (99, 100, 135), including sexual abuse (95). In fact, parents of autistic children are more likely to be emotionally and physically punitive at the child’s behavior (e.g., non-responsiveness and rigid adherence to routines) as they may perceive it as oppositional (142). Maltreatment is associated with increased dysregulated behaviors in autistic children (e.g., aggression and self-harm) (100), with those who have been abused being at greater risk of engaging in dysregulated behaviors, including suicide attempts (99). For instance, Taylor and Gotham (133) found that 90% of their sample of autistic children with high mood symptoms had experienced at least one traumatic event. Importantly, heightened exposure to trauma may predispose autistic children to develop a co-occurring BPD given that early traumatic experiences are a key risk factor for the disorder (143).

Additionally, autistic children have a 4-fold increased risk of being bullied at school compared to their non-autistic peers (144, 145) due to the misunderstanding of their atypical functioning and social difficulties (144), with those without intellectual disability being at higher risk (100). Importantly, repeated adverse experiences, including school bullying, have been shown to be associated with higher levels of distress and altered physiological arousal in adulthood (146). Recent findings by Camodeca & Nava (147) add to these results by showing that victimization strongly predicts increased physiological arousal to emotional triggers in non-autistic adults. Interestingly, this association has also been found in the case of exposure to bullying perpetrated to others (i.e., witnessing bullying without intervening) (147).

Furthermore, the heightened exposure of autistic people to adverse experiences persists in adulthood (140, 148). Indeed, autistic adults report more emotional bullying and greater sexual victimization compared to the general population (148, 149), particularly women (94, 95). Additionally, autistic adults, especially those who were diagnosed in adulthood, may suffer from a lack of social support, including from relatives who reject or misunderstand their diagnosis (150–152).

Given that chronic invalidation may be widespread in ASC (i.e., family members, school, society), autistic adults may present with high levels of internalized stigma related to their ASC diagnosis [e.g., (153, 154)], which may, in turn, contribute to high levels of autistic camouflaging, i.e., efforts of masking and/or compensating for autistic traits to ‘fit in’ in society (155, 156). Recent findings show that autistic camouflaging negatively affects mental health (e.g., depression and anxiety) (91, 93) and is associated with lifetime suicidality in autistic adults without intellectual disability (157, 158), especially autistic women (90, 91). Interestingly, autistic camouflaging is akin to self-invalidation in Linehan’s model in many ways. First, both are the consequence of invalidation in childhood (159). Second, both teach one to mistrust one’s internal states and to rely on the environment for clues on how to respond. Third, the tendency to look for external validation in both cases interferes with developing a sense of self (156). Fourth, both might be of adaptive value to avoid negative reactions such as violence and bullying (155).

#### Lack of parental scaffolding and modeling

Caregivers play a key role in helping the child learn effective emotion regulation, particularly through parental scaffolding, defined as a parent’s support of their child’s emotion regulation (115, 160). This is especially the case for autistic children, given their vulnerability to develop ED and the increased influence of parental behavior on their social and emotional development (115, 161). In autistic children, effective parental scaffolding has been associated with enhanced emotion regulation, while low parental scaffolding has been associated with ED (115, 160, 162, 163). In addition, some findings show that parents of autistic children may mainly rely on passive co-regulation strategies while providing emotional scaffolding (i.e., following the child’s lead), instead of active strategies (i.e., prompting/helping, redirection of attention, physical comfort) (162).

Additionally, studies point to parental ED as a potential contributor to ED in autistic youth (57, 97, 162, 164). Indeed, fewer parental externalizing problems (e.g., aggression, hyperactivity) have been linked to adaptive emotion regulation skills in autistic children (162). This may reflect that providing effective parental scaffolding and modeling for emotional regulation requires the parents to be able to use effective emotion regulation strategies to regulation their own emotions (164-166). For this reason, Flujas-Contreras et al. (165) investigated the impact of a clinical intervention aiming at enhancing the parents’ emotion regulation skills (e.g., mindfulness skills, problem solving and strategies for managing their children’s behavior and emotional problems) on their autistic children’s emotion regulation abilities. Unsurprisingly, both the parents’ and the children’s emotion regulation abilities improved significantly following the intervention (165). These results bring additional support to the development of parent-based interventions to enhance ER abilities in autistic children (165, 166). These early interventions may be of preventive value in helping to thwart the development of ED in autistic people since childhood.

### Illustrative case conceptualization

**Figure 2:**
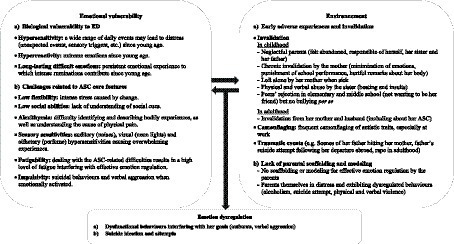
Case conceptualization of Mrs. F. based on our application of Linehan’s model to ED in ASC.

Mrs. F. is a 37-year-old woman who has a full-time job and lives with her husband and young child. Mrs. F. was diagnosed with ASC without intellectual disability at the age of 35, in addition to previous diagnoses of postpartum depression and PTSD. She was subsequently diagnosed with ADHD. Mrs. F. has experienced daily suicide ideation since childhood. As a child, she frequently reflected on ways to attempt suicide and tried once to die by stopping to eat. In adulthood, she attempted suicide twice by medication overdose. Both suicide attempts required hospitalizations in an intensive care unit. Mrs. F. does not exhibit self-harm without suicidal intent. She underwent a comprehensive psychiatric evaluation as part of her ASC diagnostic assessment, including the Mini-International Neuropsychiatric Interview (M.I.N.I.; (168, 169), for the French version). No co-occurring BPD was identified. No one else in Mrs. F.’s family is known to have received an ASC diagnosis. Mrs. F’s case formulation using our application of the biosocial model to ASC is in Figure 2 and her scores on self-reported scales measuring dimensions related to the components of the model are shown in Table 1.

**Table 1:** Mrs. F.’s scores on a battery of scales measuring the components of Linehan’s model applied to ASC.

Scale	Assessed dimension	Mrs. F.’s score	Reference value^*^	Maximum score
Autism Spectrum Quotient [AQ; (170)]	Autistic traits	44^a^	32	50
Beck Anxiety Inventory [BAI; (171)]	Anxiety	33^a^	30	63
Beck Depression Inventory-Second Edition [BDI-II; (172)]	Depression	44^a^	29	63
Difficulties in Emotion Regulation Scale [DERS; (173)]	Emotion dysregulation	124^a^	96	175
Beck Scale for Suicide Ideation [BSS; (171)]	Suicide ideation	27^a^	26	38
Eight-item General Alexithymia Factor Score [GAFS-8; (174)]	Alexithymia	51	60	40
Camouflaging of Autistic Traits Questionnaire [CAT-Q; (175)]	Autistic camouflaging	108 ^a^	100	175
Sensory Processing Sensitivity Questionnaire – Sensory Sensitivity Subscale [SPSQ; (176)]	Sensory sensitivities	7.75 ^a^	5.2	10
Emotional Vulnerability-Child Scale [EV-Child; (177)]	Emotional vulnerability in childhood	119 ^a^	62	132
The Childhood Trauma Questionnaire—Short Form [CTQ-SF; (178)]	Childhood trauma			
	Emotional abuse	20 ^a^	13	25
	Physical abuse	5	10	25
	Sexual abuse	5	8	25
	Emotional neglect	17 ^a^	15	25
	Physical neglect	12 ^a^	10	25

AQ: clinical cut-off (170); BAI: cut-off for severe anxiety (171); BDI: cut-off for severe depression (172); DERS: cut-off for severe ED suggested by Neacsiu et al. (179); BSS: cut-off used by van Spijker et al. (180); GAFS-8: cut-off for high to very high alexithymia in a sample from the general population (174); CAT-Q: cut-off used by Cook et al. (181); SPSQ: mean score in a sample of women from the general population (182); EV-Child: mean score in a sample of female students (177); CTQ-SF: cut-off scores for moderate to severe from Bernstein and Fink (183). ^a^Score above the reference value.

#### Emotional vulnerability

Mrs. F. reports that several ordinary events can trigger distress and intense reactions (e.g., bursting into tears), resulting in a wide range of situations as potential crisis triggers (e.g., «I do not understand why I can get so distressed over something that is not really important»). This is akin to hypersensitivity to emotional cues relative to Linehan’s model (1993). She also describes feeling intense and long-lasting emotions fueled by ruminations (e.g., «It [the emotion] feels like a geyser», «It [the emotion] stays there for a long time […] stagnant», «It goes round and round in my head»). This is supportive of the two additional facets relative to emotional vulnerability in Linehan’s model (1993): i.e., emotional hyperreactivity and slow return to baseline when facing difficult emotions. Mrs. F. reports dealing with these emotional difficulties since a very young age. These elements along with Mrs. F.’s self-reported problems in understanding her emotions (i.e., alexithymia) indicated that it was crucial to include these elements into the psychoeducation on the biological vulnerability component of the model. Indeed, these elements, along with the ASC-related difficulties highlighted below, probably contribute to the high level of distress Mrs. F. experiences on a daily basis. This understanding is essential to increase emotional awareness and decrease self-invalidation (e.g., «I do not understand why I can get so distressed over something that is not really important») and shame, which are, respectively, a prerequisite for effective emotion regulation and a motivational factor.

Regarding ASC-related factors associated with ED included in our application of Linehan’s biosocial model to ED in ASC, Mrs. F. reports that, due to her need for sameness, last-minute changes provoke intense anxiety. Becoming a mother at the age of 32 has been a major additional source of stress, as her child’s changing needs and reactions are a source of unrelenting unexpected events and sensory discomfort (e.g., her child’s crying). Difficulties in reading social cues are a major source anxiety, as they are associated with doubts over how to interpret and react to others’ behavior. In addition, Mrs. F. has auditory (noises), visual (neon lights) and olfactory (perfume) hypersensitivities that cause overwhelming sensory experiences (e.g., «It is an invasion [...] it can cause extreme discomfort», «I struggle to put it [the sensory stimulation] aside and be available for the rest of the things»). Mrs. F. also describes having difficulties identifying and describing her bodily experiences, as well as understanding what causes her physical pain. This is especially the case when she is in an emotional crisis (e.g., «When I am overwhelmed, there is no access to anything [in her body and mind], I am just in survival mode»). In extreme cases, Mrs. F.’s impulsivity can lead to verbal aggression or unplanned suicidal behaviors, which was the case in her first suicide attempt. Dealing with the ASC-related difficulties and camouflaging them result in a high level of fatigue. This interferes with her ability to regulate her emotions effectively (e.g., «Compensating for sensory overload, camouflaging, social interactions and, also, managing my emotions… it is exhausting! »). Camouflaging in particular is described as extremely costly and exhausting (e.g., «I do it deliberately to modify my behavior […] it is there all the time and it is exhausting»). Her responsibilities as a mother also add to the daily fatigue, as she needs to attend to the constantly changing needs of her child. Highlighting the ASC-related difficulties and adding them to the biosocial model allowed to specifically target them, especially in individual sessions. Indeed, as highlighted in a first-person account of an autistic person who benefitted from DBT (184), DBT therapists need to consider the specific needs and motivational factors of autistic clients to increase the pertinence of DBT. Here, this means to be aware of ASC-related features that are likely to contribute to ED, integrate them in the biosocial model, as well as in the targets and goals of the therapy. In addition, therapists’ destigmatizing attitudes toward ASC draw upon this conceptualization, as it aids to validate the difficulty to cope with these challenges on a daily basis (e.g., adapt to a non-autistic world), to teach to self-validate instead of self-stigmatize and camouflage, to provide targeted psychoeducation (e.g., on autistic camouflaging and its impact on mental health), to help identify and label emotions, but also problem-solve (e.g., in relation to sensory triggers).

#### History of invalidation and adverse events

Mrs. F.’s parents had alcohol use disorder. She reports feeling abandoned in her childhood, unable to rely on her parents. During her childhood and adolescence, she witnessed repeated scenes of physical violence perpetrated by her father on her mother, and, at times, by her sister on her mother. Some of these scenes were traumatic. Emotional abuse was frequent, as Mrs. F.’s mother repeatedly told her «no one will love you, you’ll end up alone» or «go look in the mirror how ugly you are» while Mrs. F. was crying. She was an excellent student throughout her school years. In response to her high grades, her mother used to say «you could have done better». Mrs. F. reports that her mother did not care for her even when she was sick, always prioritizing work. Mrs. F. also reports that her sister was physically and verbally violent towards her, hitting and insulting her. Her mother’s invalidation (e.g., punitive and oversimplifying behaviors) continued into adulthood. She explains that her father was the only person she felt understood by. However, her father attempted suicide after she left home to live abroad. Before her departure, he told her that he would attempt suicide if she left. Mrs. F. reports that she still feels guilty over her father’s suicide attempt.

Mrs. F.’s parents exhibited dysregulated behaviors (alcohol use disorder, physical and verbal violence, and suicide attempts) indicative of a great psychological distress and major difficulties to regulate their emotions. Thus, Mrs. F.’s parents were not able to provide her with the necessary emotion regulation scaffolding and modeling. On the contrary, their own difficulties were a source of recurrent invalidation and trauma. In addition to the trauma related to events in her family, Mrs. F. was a victim of rape as a young adult, and subsequently developed a PTSD related to this event.

At school, Mrs. F. reported feeling isolated. Nevertheless, she reported that school was “a safe haven” because it was a structured environment, where she found intellectual fulfillment, and support from the teachers. In middle school, she was the target of bullying from peers. She had difficulty integrating groups of friends (e.g., «I did not have the codes of how things were done») and felt rejected.

In later years, Mrs. F. has experienced high levels of invalidation regarding her ASC diagnosis, both from her family and her husband, e.g., «you are just lazy», leading to increased anger, shame and sadness.

The psychosocial factors highlighted here were key to better understand Mrs. F.’s developmental environment, as well as its potential effects on ED and on her overall mental health. Specifically, they helped to identify predisposing factors that seem to have contributed to self-invalidating behaviors [i.e., a secondary target in DBT involved in ED; (5)] and, more broadly, to ED – e.g., repetitive punitive invalidations and lack of scaffolding and modeling of effective emotion regulation from her parents. Given the transaction between the invalidating environment and her emotionally vulnerable temperament, including her ASC-related difficulties, it is understandable that she felt and reacted the way she did. This knowledge, inherent to the dialectical perspective of the biosocial model (5), was crucial for the therapist to validate the client and to teach her to self-validate. In addition, this allowed the therapist to provide psychoeducation on the possible link between adverse events (e.g., parents’ dysregulated behaviors, lack of emotional scaffolding) and current emotional difficulties (in transaction with biological factors), including suicidal behavior. According to the DBT framework and conceptualization (5), Mrs. F. was in stage 1 of DBT, that is, she presented with behavioral dyscontrol (e.g., life-threatening behaviors). It is only in stage 2, once dysregulated behaviors are no longer present, that PTSD and the sequelae of traumatic and invalidating experiences may be directly targeted.

## Discussion

This article aimed at applying and extending Linehan’s biosocial model (5) to ED in autistic people across the lifespan. This is of particular interest as ED is prevalent in this population (57, 185) and seems to be involved in the high rates of self-harm and suicidality (40, 41). Consequently, DBT is an emerging topic in the field of interventions targeting ED in ASC, with promising preliminary results (50, 51). However, no studies so far had focused on the utility of Linehan’s biosocial model, which underlies DBT for BPD, in ASC.

Our review and application to the case of an autistic woman suggest that ED in ASC encompasses factors related to both biological and psychosocial risk factors as conceptualized in the BPD framework, although in both domains ASC-specific factors might be involved. Indeed, in addition to the biological vulnerability similar to BPD (i.e., hypersensitivity, hyperreactivity and slow return to emotional baseline) (105), ToM peculiarities, sensory sensitivities, lack of cognitive flexibility, change-related anxiety and RRBs have been associated with ED in ASC (39, 51). Alexithymia, prevalent in ASC, has also been reported to be linked to ED in autistic adults (41), especially in autistic women (123). It is worth noting that ASC-related difficulties may interfere directly with the ability to self-regulate (32, 39, 51) but also contribute to high levels of anxiety and fatigue making emotion regulation costly for autistic people (39, 51, 117). Such is the case for Mrs. F., whose autistic features (e.g., sensory hypersensitivity, hypervigilance regarding social rules and how to behave in social situations) are both involved in her emotional vulnerability and in the costs of real-life use of adaptive emotion regulation skills. We note, however, that people with BPD might present with autistic-like features, including sensory hypersensitivities (187) and ToM peculiarities (188). Therefore, our findings support the application and extension of Linehan’s model to ASC, but it also highlights under-researched topics in BPD. Indeed, it is likely, for example, that sensory particularities may also play a role in ED in people with BPD, as this has been shown to be the case in the general population (189).

Regarding psychosocial risk factors, our review suggests that, similar to BPD and other psychological conditions (4, 28, 29), invalidating experiences seem to contribute to the emergence of ED also in autistic people. In fact, findings report that autistic children are highly exposed to different early stressful and traumatic experiences (e.g., physical and emotional maltreatment from caregivers and school bullying), especially because of their atypical functioning that cause misunderstanding and rejection from others (131, 132, 139, 187). Autistic girls seem to be particularly vulnerable to experience these adverse events (134, 135). Importantly, adverse experiences have been associated with co-occurring psychopathology and/or the worsening of difficulties related to ASC in childhood (133, 141). As in BPD, these experiences have been associated with self-harming behaviors with or without suicidal intent in autistic children (99, 100), particularly in those who have been sexually abused (99). In adulthood, these experiences have been associated with numerous co-occurring disorders such as mood and anxiety disorders, PTSD (131), and BPD (143, 190).

Beyond the impact of adverse experiences, given the potential transactional link between biological and environmental factors of ED in ASC, it is likely that the specific needs of the autistic child might differ from those of the child at risk to develop BPD. For instance, autistic children might need increased parental scaffolding and modeling to learn effective emotion regulation skills than children who will develop BPD (115, 165). The necessary adjustments of the caregivers (e.g., teachers, parents) can be promoted by an early diagnosis of ASC (191) and enhancing the parents’ emotion regulation skills (165).

In addition, our review and extension of the biosocial model to ASC includes excessive autistic camouflaging as a form of self-invalidation resulting from internalized invalidation from others (192). In addition to being costly, the self-invalidation associated with autistic camouflaging might be detrimental to the development of adaptive emotion regulation skills as well as to the sense of self and self-acceptance in autistic people (156, 193). Importantly, recent studies found a strong negative association between autistic camouflaging and lifetime suicidality in autistic adults, especially autistic women (90, 91, 157). The latter finding can be explained by several factors. Indeed, autistic women, especially those without intellectual disability, are diagnosed later than men (92). Relatedly, greater expectations for adolescent and adult autistic women to engage in adaptive social communication and behavior are more prominent (91). This may, in turn, be involved in the enhanced use of compensatory behavior to mitigate social challenges and mask autistic traits in autistic females, i.e., camouflaging (91, 192). Therefore, if autistic women, especially those who are undiagnosed, are more likely to mask their mindreading and overall social difficulties, this may promote their social inclusion (194). However, they probably lack the social skills that might make them less vulnerable to societal invalidation towards women in general, including sexual violence (94, 95, 195). Mrs. F.’s case illustrates the impact of late diagnosis (at the age of 35) and the resulting autistic camouflaging to “try to fit in” since childhood. We speculate that an early diagnosis could be beneficial in several ways. For example, it could foster the understanding of one’s own functioning, prevent self-invalidation and enhance self-acceptance in autistic people. In autistic women, earlier diagnosis could also be of preventive value in relation to sexual violence, enabling access to targeted sexual education and assertiveness programs (195).

Moreover, regarding the overlap in biosocial correlates of ED between ASC and BPD, it appears crucial to expand our knowledge of ED and its mechanisms in both diagnoses. Thus, we suggest considering the following points of potential differentiation in future studies. First, people with BPD might present with autistic-like features, such as sensory hypersensitivities (187) and ToM peculiarities (188). Thus, comparative studies are needed to investigate the extent to which the ASC-related factors specifically contribute to ED in ASC relative to disorders with ASC-like features such as BPD. We also suggest to further investigate how these factors may interact with each other and contribute to ED. For instance, recent findings suggest an association between high sensory sensitivity (i.e., low sensory threshold and ease of excitation) and alexithymia in non-autistic young adults, with this interaction impacting emotion processing and regulation (196). Second, ED in BPD is significantly involved in interpersonal problems (e.g., conflicts, physical/verbal violence) due to mood swings and chronic fear of abandonment (62, 197). In ASC, relationships are not likely to be affected in the “stormy” way found in BPD, as social difficulties are rather related to poor social abilities that makes it difficult to bond with others (198). It seems therefore relevant to investigate the impact of ED on relationships in both BPD and ASC to potentially highlight distinctions in ED between the two diagnoses. Third, in BPD, ED is strongly linked to affective instability in interpersonal contexts (199). In ASC, by contrast, ED seems to arise from the interaction between ASC traits and contextual factors (e.g., invasive sensory stimuli, changes in the environment/planning) (38, 39). Thus, it seems relevant to further investigate whether and how ED might be more related to widespread context cues in ASC compared to BPD. Fourth, to date, evidence supports the role of emotional vulnerability and invalidation separately in the development of ED in ASC. Thus, the transaction between the two is yet to be empirically tested in ASC. Fifth, previous findings have reported differences in the development of personality styles between ASC and BPD (200). Hence, it seems relevant to explore whether and how the factors inherent to the development of personality in BPD contribute to ED relative to ASC (201).

Finally, we acknowledge that the paucity of data on ED and its mechanisms in ASC might have limited the potential determinants of our application of Linehan’s model. Nevertheless, given the increasing awareness of the impact of ED on autistic individuals’ mental health, our application has the advantage of providing a pragmatic model that can inform the delivery of psychological treatments for this population. Additionally, our application of the model to ASC may foster new and much needed research on the biosocial mechanisms of ED in ASC. For instance, as a growing body of research has shown that autistic women are at greater risk for severe ED (61) and suicidality than men (86). Hence, it seems crucial to consider this discrepancy in future studies by systematically exploring cross-gender (including transgender and gender non-conforming individuals) differences in the implication of the determinants of ED in ASC. Moreover, our application might provide clinicians with a comprehensive framework of ED in ASC, enhancing the systematic assessment of traumatic and invalidating experiences in autistic clients. The latter include different layers of invalidation, which might be related to intersectional factors (e.g., gender) involved in family invalidation, peer invalidation but also societal discrimination leading to self-stigma and self-invalidation (94). Finally, our application of the model may inform psychological treatments targeting ED, especially DBT, the psychotherapy that has amassed the most evidence in the treatment of ED in BPD (5, 12). As DBT was developed based on Linehan’s biosocial theory (5), applying and extending this theory to ED in ASC may foster the adaptation of DBT to treat ED in autistic people, especially since preliminary data on its feasibility and effectiveness in autistic adults are promising (49, 50). This is of particular importance as evidence-based interventions targeting ED in autistic individuals are lacking, especially for adults (202), and that ED might be associated with self-harm and suicidal behaviors in this population (40, 41), especially autistic adults without intellectual disability (86). Furthermore, our application of the Linehan’s biosocial model to ASC might promote specific adaptations to autistic people, increasing the acceptability and efficacy of DBT applied to this population (184). For example, increased scaffolding and modeling from the therapist and the use of a psychoeducational biosocial model that integrates the ASC specificities highlighted here might be particularly valuable to this aim.

## Author contributions

DB: conceptualization, methodology, investigation, and writing – original draft. LW: conceptualization, methodology, supervision, and writing – review and editing. All authors contributed to the article and approved the submitted version.

## Footnotes

^1^Note on terminology: Throughout the manuscript, we use the term “autistic people” (identity-first), rather than “people with autism” or “people with autism spectrum disorder,” as this was the terminology explicitly favoured by the majority of the autistic participants of a large-scale survey (30). We also use “autism spectrum condition” instead of the DSM-5 term of “autism spectrum disorder (ASD)” to be respectful to those on the spectrum who feel that the term “disorder” is stigmatising, whereas the term “condition” acknowledges both the difficulties and the differences and strengths in autistic people (31).
